# Platelet-Rich Plasma for Patients with Olfactory Dysfunction: Myth or Reality? A Systematic Review

**DOI:** 10.3390/jcm13030782

**Published:** 2024-01-29

**Authors:** Antonio Moffa, Domiziana Nardelli, Lucrezia Giorgi, Simone Di Giovanni, Luca Carnuccio, Carmen Mangino, Peter Baptista, Michele Vacca, Manuele Casale

**Affiliations:** 1 Integrated Therapies in Otolaryngology, Fondazione Policlinico Università Campus Bio-Medico di Roma, 00128 Rome, Italy; 2 School of Medicine, Università Campus Bio-Medico di Roma, 00128 Rome, Italy; 3 Unit of Measurements and Biomedical Instrumentation, Department of Engineering, Università Campus Bio-Medico di Roma, 00128 Rome, Italy; 4 Department of Otorhinolaryngology, Clinica Universidad de Navarra, 31008 Pamplona, Spain; 5 ENT Department, Al Zahra Private Hospital Dubai, Dubai 23614, United Arab Emirates; 6 Transfusion Medicine and Cell Therapy, Fondazione Policlinico Università Campus Bio-Medico di Roma, 00128 Rome, Italy

**Keywords:** olfactory dysfunction, platelet-rich plasma, smell loss, anosmia, COVID-19

## Abstract

Background: With promising outcomes, platelet-rich plasma (PRP) has recently been suggested as a treatment for olfactory dysfunction (OD). Methods: Clinical studies utilizing PRP in OD caused by COVID-19, trauma, anesthetic exposure, viral infection, and chronic rhinosinusitis were included in a systematic review. Results: Ten clinical studies were qualitatively analyzed. Six of these studies used the PRP for OD caused by COVID-19, one on OD after functional endoscopic sinus surgery, and three on post-infectious or post-trauma OD. The population included 531 patients, ranging in age from 15 to 63. Conclusion: The use of PRP may be a risk-free and efficient therapeutic option with very encouraging outcomes. Indeed, it enhances olfactory perception in patients who not only exhibit COVID-19 infection aftereffects, but also in those who have lost their sense of smell due to trauma, rhinosinusitis, rhinitis, or even surgery. To evaluate the PRP’s therapeutic benefits in OD patients and to compare the efficacy of different therapeutic protocols with regard to treatment schedules, there is an urgent need for focused controlled trials.

## 1. Introduction

Olfactory dysfunction (OD) is a common disorder that has a negative impact on quality of life and is thought to affect up to 20% of the global population. It is also associated with higher rates of morbidity and death [1]. OD can be defined as quantitative when the strength of odors is affected, or qualitative when the quality of odors is changed or there is the perception of smell in the absence of an odor stimulus. Qualitative disorders, such as parosmia, often entail qualitative changes perceived as unfavorable. Qualitative changes are rarely found alone, as they often present in combination with a quantitative disturbance.

OD can be divided into three main categories based on the anatomical location of the lesion: conductive, sensorineural, and central. Yet, anatomical classification can be restrictive, as the three abovementioned categories are not mutually exclusive, which may result in underestimating the underlying pathophysiology. Therefore, OD can be further classified upon its putative underlying etiology into post-infectious olfactory dysfunction (PIOD), which branches into COVID-19-associated PIOD (C19OD); olfactory dysfunction secondary to sinonasal disease; post-traumatic olfactory dysfunction (PTOD); olfactory dysfunction associated with neurological diseases; olfactory dysfunction associated with exposure to drugs/toxins, congenital olfactory dysfunction; olfactory dysfunction associated with aging (presbyosmia); and iatrogenic/comorbid and idiopathic olfactory dysfunction [2]. Between 34.6% and 62.0% of patients infected with COVID-19 are estimated to suffer from C19OD, rendering it a highly prevalent symptom characterizing the clinical picture of COVID-19 [3,4]. Interestingly, about 11% of patients report a gustatory dysfunction associated with C19OD, either perceived as two distinct entities or as a unified symptom [5]. The presence of a “loss of smell” also gained a strong positive predictive value (61%) in forecasting COVID-19 positivity [4], especially in population screening for asymptomatic COVID-19 carriers. In fact, the sole presence of anosmia in an otherwise asymptomatic individual proved to be an indicator of positive carriage [6].

To date, there are no long-term, effective treatments for OD. This is mainly attributable to a lack of high-level evidence in the literature due to lack of funding, insufficient participants, and inherent methodological and/or hypothesis-driven differences that prevent the generalization of results. Yet, the impact of the COVID-19 pandemic has drawn major efforts and attracted funding for OD treatment.

According to the position paper on OD published in 2023, systemic (short-term) and/or intranasal (long-term) corticosteroids should be prescribed in patients with OD secondary to chronic rhinosinusitis (CRS), severe allergic rhinitis, and other inflammatory conditions [2]. When intranasal corticosteroids are used, a delivery mechanism that can reach the olfactory cleft (rinses) would be recommended. Moreover, olfactory training can be recommended in patients with olfactory loss due to several etiologies, including PIOD and PTOD. However, this treatment requires further evaluation in patients with sinonasal inflammatory disease and neurodegenerative diseases.

Functional endoscopic sinus surgery for olfactory loss caused by the CRS disease spectrum should be undertaken in line with existing guidelines and is not recommended in the absence of CRS. In severe CRS with nasal polyposis, biologic treatment appears to improve OD. Among them, dupilumab seems to be the most effective [2].

Platelet-rich plasma (PRP) has recently been proposed for OD treatment with encouraging results. PRP is an autologous or homologous biologic product obtained from freshly drawn blood containing a high concentration of platelets in a small plasma volume. PRP is endowed with anti-inflammatory and pro-regenerative properties, including upregulation of growth factors, such as transforming growth factor-beta, endothelial growth factor, vascular endothelial growth factor, nerve growth factor, and insulin-like growth factor, contained in alpha granules [7]. Collectively, these factors promote angiogenesis, cell proliferation, differentiation, and survival. The latter properties have been exploited to enhance tissue healing and regeneration in a vast body of clinical and surgical settings since the 1970s. Moreover, PRP can foster axon regeneration and neuroregeneration [8].

Studies on animal models have demonstrated that growth factors and stem cells can successfully treat anosmia and regenerate the olfactory neuroepithelium, which is amenable as a therapeutic target for PRP neuroregeneration [8,9]. Indeed, due to its growth factor and neurotrophic-rich nature, PRP has shown promising results in treating anosmia in animal models [10].

In clinical practice, PRP has recently gained popularity in otolaryngology [11]. Its uses range from fostering wound healing after myringoplasty [12,13], tonsillectomy [14,15], rhinoplasty [16,17], endoscopic sinus surgery [18,19], vocal fold scar, atrophy, and sulcus vocalis [20,21] to treatment of sensorineural hearing loss [22,23,24] and endoscopic CSF leak repair [25]. In the 2023 position paper on OD, PRP is listed among the treatments of qualitative OD [2].

To our knowledge, only systematic reviews concerning the use of PRP in C19OD settings are available in the literature [26,27]. Therefore, the effects of PRP application in OD subtended by other rhino-sinus pathologies have not been reviewed yet. This systematic review aims to revise the currently available literature on all the use of PRP in OD caused by CRS, trauma, anesthetic exposure, or viral infection and also COVID-19.

## 2. Materials and Methods

### 2.1. General Study Design

The study was designed following the recommendations of the Centre for Review and Dissemination’s Guidance for Undertaking Reviews in Health Care and is being reported in adherence with the Preferred Reporting Items for Systematic Review and Meta-Analyses (PRISMA) statement [28]. This systematic review was not registered in the International Prospective Register of Systematic Reviews (PROSPERO) or anywhere else.

### 2.2. Search Strategy and Selection of Studies

Studies published until November 2023 were identified from PubMed, SCOPUS, EMBASE, Web of Science, and Cochrane databases. An example of a search strategy used in PubMed/MEDLINE is: “platelet-rich plasma OR PRP OR platelet rich plasma OR platelet-rich plasma injection” and “Olfactory dysfunction OR anosmia OR hyposmia OR parosmia” and “COVID-19 olfactory dysfunction” and “Functional endoscopic sinus surgery OR FESS.” All the searches were adjusted to fit the specific requirements for each database, with a cross-reference search to minimize the risk of missing relevant data.

### 2.3. Inclusion/Exclusion Criteria

According to the PICOS acronym [29], we included the studies with the following characteristics: patients (P), patients seeking treatment with PRP for OD (anosmia, hyposmia, or parosmia) due to COVID-19, trauma, infection, anesthetic exposure, and CRS; intervention (I), in-office and peri-operative procedures using PRP injection into the nasal fossae; comparison (C), pre- and post-treatment; outcome (O), objective tests (e.g., threshold, discrimination, and identification test—TDI, the smell detection threshold—STC and the smell identification test—SIC), self-reported tests (e.g., VAS for parosmia, olfactory dysfunction questionnaire—ODQ) and the side effects; and study design (S), both prospective and retrospective cohort studies.

The exclusion criteria were: (1) studies not in English; (2) case reports, reviews, conference abstracts, letters, and pediatric studies; (3) studies with unclear and/or incomplete data; (4) studies conducted on animal models; (5) studies evaluating the effects of PRP application where olfactory function was not an overtly assessed outcome, and (6) studies where preparations other than PRP were employed. No publication date restriction was imposed.

### 2.4. Data Extraction and Data Analysis

Two reviewers (AM, DN), working independently, screened all abstracts and titles for candidate studies and discarded studies unrelated to the use of PRP in OD. The full-text version of each publication was assessed, and those whose content was judged not strictly related to this review’s subject were excluded. Data extraction of the studies included the population demographics and baseline characteristics, details on intervention and control conditions, study designs, and outcomes.

A qualitative synthesis analysis was performed, comparing pre-treatment and post-treatment values and/or between post-treatment and control or placebo outcomes during the follow-up period.

### 2.5. PRP Extraction and Application

The following protocol for PRP extraction and application was used in the studies included in the review. The first step consisted of blood extraction (20 mL) into a tube with sodium citrate (SC) anticoagulant and the isolation of PRP through a 10 min centrifugation at 4200 rpm. The supernatant was gathered into a 10 mL syringe. Then, local anesthesia was performed with Xylocaine 10% spray 2 min after the injection of xylometazoline chlorhydrate drops into the nasal fossae. The injection was performed through a 0° rigid optic to guide the needle direction. To provide better access to the anatomical region, the needle may be bent to 30°. Several points of 0.2–0.5 mL were performed in the middle turbinate and the nasal septum regarding the head of the middle turbinate and in the anterior part of the region. In anatomic deviation, the injection was performed closest to the olfactory cleft. The procedure was similarly performed on the contralateral side. Patients were observed for 15 min post-procedure for potential adverse effects and ultimately discharged [30].

## 3. Results

**Figure 1:**
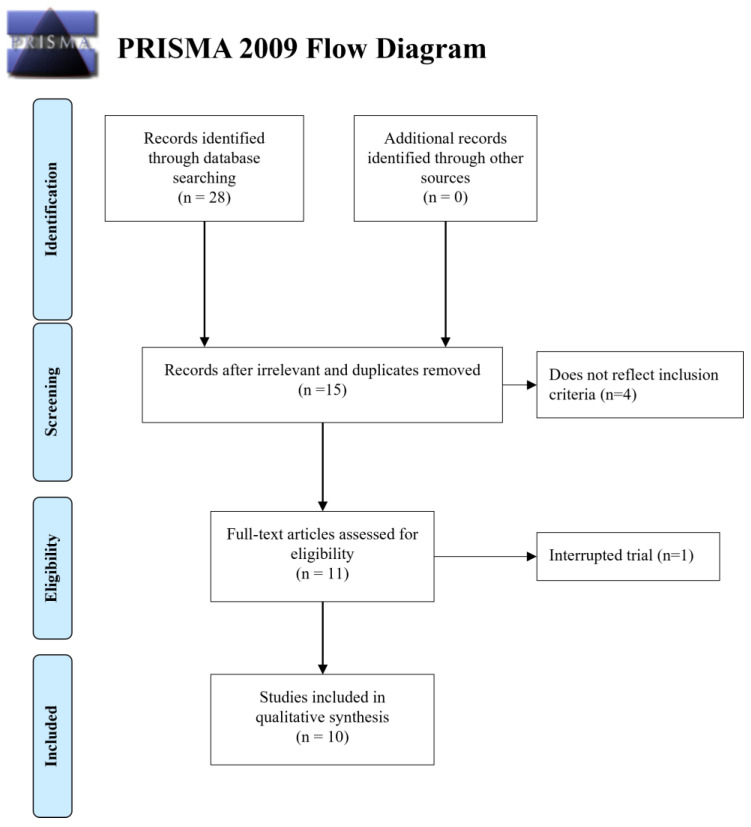
Flowchart outlining the paper selection process of the systematic review (based on PRISMA guidelines).

Search criteria returned 28 articles, and then 13 were removed as irrelevant or duplicates. These were screened, and 5 articles were excluded, resulting in 10 articles fulfilling the inclusion criteria, all published in the last seven years. A flow diagram in Figure 1 (PRISMA Flow Diagram) depicts the selection process. All the original articles included were prospective clinical studies. The population in the included studies consisted of 531 patients aged between 15 to 63 years old. The baseline characteristics of the studies included are shown in Table 1, and a further description of the studies conducted in the reports can be found in Table 2.

### 3.1. PRP and Olfactory Dysfunction

Mavrogeni P. et al. [31] were the first to investigate the role of PRP in OD settings. Their pioneering study involved five patients affected by anosmia (one suffered from injury, and four had virus rhinitis) for at least three months. They all received three injections containing 1 mL of PRP spaced by a 4-week interval and a further fourth injection after three months. After the third and fourth injections, four out of five patients stated that “their smell came back,” while the remaining patient reported that he could “smell a lot but not everything”. Moreover, two patients who had even lost taste could tell sweet, sour, bitter, and salty apart.

Yan C.H. et al. [32] and Shawky M.A. et al. [33] enquired about the efficacy and safety of PRP injections in patients affected by OD lasting between 6 and 11 months, with no evidence of sinonasal inflammatory disease and no improvement after olfactory training or topical steroid rinses were enrolled. Differently from the standard protocol, they used the GS30-PURE II Protocol A (Emcyte, Ft Myers, Florida) to isolate PRP products with high platelets, low granulocyte counts, and minimal erythrocytes. In this case, 20 mL of blood was drawn, added to 5 mL of SC anticoagulant, and centrifuged at 4200 rpm for 1 min, upon which the supernatant was aspirated and re-centrifuged at 4200 rpm for 5 min. The subsequent supernatant platelet-poor plasma was discarded until 2 mL of PRP remained. The PRP was finally drawn up into two separate 1 mL syringes. The first study [32] included seven patients who received a one-time submucosal injection containing 1 mL of PRP per each olfactory cleft. The TDI using the Sniffin’ Sticks^®^ Test was administered at baseline, 1 month, and 3 months post-injection. All patients reported a subjective improvement of their smell shortly after injection. At three months post-injection, there was an overall significant improvement in TDI (*p* = 0.026): two patients with functional anosmia (TDI < 16) did not improve significantly, while five patients with hyposmia (TDI 16–30) showed an improvement of 60%, achieving normosmia (TDI > 30). No adverse effects were reported. In the Shawky M.A. et al. study [33], 27 patients received a 1 mL PRP injection into the olfactory cleft, while 27 received two injections three weeks apart. The Q-Sticks test was employed to measure olfaction and was administered at baseline, 1 month, and 3 months after the last injection. Similarly to the findings of Yan et al. [32], all patients reported a subjective improvement shortly after injection. At three months post-injection, significant improvement was observed in 16 patients after single injection (*p* < 0.001) and in 19 patients after double injection (*p* < 0.001). No adverse outcomes were reported.

### 3.2. PRP and COVID-19 Olfactory Dysfunction

Steffens Y. et al. [34] investigated the usefulness and safety of PRP injection in 56 patients with C19OD, adopting the same protocol used by Yan et al. and Shawky et al. [32,33]. Thirty patients were allocated to the PRP group, while 26 were assigned to the control group, receiving simple olfactory training. The mean duration of the olfactory disorder was 10.8 ± 2.5 months in the PRP group and 9.7 ± 3.4 months in controls. Patients in the intervention group received one PRP injection containing 1 mL of PRP in each olfactory cleft. At one month post-PRP injection, the mean TDI scores significantly improved by 6.7 points in the PRP group (*p* < 0.001), the mean self-assessment of improvement in olfactory function (linker scale from 0 (none) to 3 (strong)) was 1.8 (mild-to-moderate) in the PRP group, which was significantly higher than in the control group (linker scale score: 0.3, *p* < 0.001). No adverse events were reported in the study.

El Naga H. et al. [35] evaluated the effects of PRP on 60 patients affected by post-COVID olfactory parosmia. All patients were non-responders to a 3-month course of olfactory training, topical steroids, omega-3, vitamin B12, and zinc supplements for six months. They followed the protocol proposed by Perez A.G.M. et al. [40], consisting of an 8.5 mL blood draw by venipuncture on the cubital vein, and 1.5 mL of acid citrate dextrose was added. Blood was never cooled. The collected tubes were centrifuged at 800 rpm for 10 min to provide a “soft” spin. Platelet-containing supernatant plasma was transferred to a new sterile tube, without anticoagulant, and received a further “hard spin” at 2000 rpm. The lower third of the tube contained PRP. In total, 30 patients were randomly allocated to the PRP group and 30 patients to the control group. The PRP group patients received three PRP injections in the olfactory cleft at three-week intervals, while the control group maintained the pre-study treatment. Both were assessed one month after treatment cessation, using a VAS (0–10) for parosmia, where a value of 0–1 indicated complete improvement. There was a highly significant improvement in both groups (3.33 in the PRP group vs. 7.43 in the control group), with a significant difference in favor of the PRP group (*p* = 0.002).

Yan C.H. et al. [36] evaluated the use of PRP in treating prolonged COVID-19-related smell loss. Twenty-six patients with an objectively measured smell loss were recruited: 18 received PRP application and 12 were allocated to placebo. The average duration of OD was 8.6 months for the placebo group and 8.9 months for the PRP group. PRP group patients received three submucosal injections at two sites within each olfactory cleft, containing 1 mL of PRP, while the placebo group received three sterile saline injections. Olfaction was evaluated at baseline and three months post-injection, resulting in improved olfaction (TDI) in the PRP group compared to a placebo of 3.67 points. There was a more significant improvement in smell discrimination following PRP (*p* = 0.004), but no difference in smell identification was found (*p* = 0.239), nor in subjective scores (*p* = 0.167). No adverse events were reported.

Similarly, Lechien J.R. et al. performed two studies [37,38]. In the first one [37], they enrolled 87 patients with anosmia, hyposmia, or parosmia, with a mean duration of 15.7 months. All patients received one injection containing 1 mL of PRP; therefore, there were no controls in this study. The injection was evaluated as somewhat or moderately painful by 41 and 22 patients, respectively. The adverse events included transient epistaxis (N = 31), parosmia related to xylocaine spray (N = 10), and vasovagal episode (N = 2). Between the baseline and two months after the injection, the mean ODQ and TDI scores significantly improved (*p* = 0.001 and *p* = 0.009, respectively). The improvement in smell was seen after an average of 3.6 ± 1.9 weeks. Subsequently, the same group [38] further investigated the effects of PRP injection on 81 patients affected by C19OD, offering a comparison with a control group of 78 untreated patients in a multicenter controlled study. The OD duration was 15.7 and 11.0 months in the PRP and control groups, respectively. Overall, 65 PRP patients reported subjective smell improvement after a mean duration of 3.4 ± 1.9 weeks. Parosmia, life quality statement, ODQ subtotal, and total scores significantly increased at ten weeks in the PRP group (*p* = 0.001). Conversely, the ODQ score did not change over time, while TDI scores significantly increased in controls at 10-week follow-up. The 10-week TDI and ODQ scores were significantly better in the PRP group compared to controls (*p* = 0.001).

Evman M. et al. [39] investigated the efficacy of PRP injection in the olfactory cleft of patients affected by C19OD lasting more than one year, unresponsive to common treatments. The study included 12 PRP and 13 control patients, who were followed without additional treatment. Patients were assessed at baseline and 1 month post-injection with the Connecticut Chemosensory Clinical Research Center olfaction test, consisting of a smell detection threshold (STC) and a smell identification test (SIC). In the PRP group, the mean STC increased from 5.6 to 6.5, while the mean SIC increased from 11.4 to 15.2. In the control group, STC increased from 5.7 to 5.8, and SIC increased from 11.2 to 11.9. A significant difference in STC (*p* = 0.037) and SIC (*p* < 0.001) was found across the two groups, concluding that a significant improvement in olfactory threshold values was observed in the PRP group when compared to controls. No adverse events were reported.

### 3.3. PRP and Functional Endoscopic Sinus Surgery (FESS)

Goljanian Tabrizi A. et al. [19] performed PRP application under general anesthesia during endoscopic sinus surgery, recruiting 48 patients with sinonasal polyposis and OD lasting longer than 3 months. Of these, 27 patients were allocated to receive PRP, while 21 patients were assigned to placebo (saline injection). Patients were assessed using the Iran Smell Identification Test (I-SIT) at baseline and 3 months post-surgery. The authors observed a significant improvement in both patients receiving a PRP injection (*p* < 0.001) and in the control group (*p* < 0.001), but no statistically significant differences in the improvement between the two arms were found (*p* = 0.802).

## 4. Discussion

COVID-19 infection revived the long-standing difficulty of treating patients affected by OD, both in its quantitative and qualitative forms, which has always represented a challenge for the ENT in clinical practice.

As evidenced by the studies in this review, PRP application may be a safe and effective procedure, portending highly promising results. In fact, it appears to improve olfaction not only in patients presenting with sequelae of COVID-19 infection but also in those who have lost their ability to smell after enduring a trauma, after rhinitis or rhinosinusitis, or even as a result of a surgical procedure. The safety of PRP use has been widely established in the literature and further confirmed in the articles mentioned above, where no major adverse events were reported. Given that PRP is an autologous biological product harvested from the patient’s blood, it eliminates the concern of rejection or disease transmission and the need for immunosuppression. It also poses an advantage in terms of costs since injections are performed in-office with the use of local anesthesia, and one injection alone has been demonstrated to bring significant improvement to most patients. Moreover, the possibility of delivering PRP selectively to the nasal fossae in a topical application denotes a major upgrade from systemic corticosteroids, which are often indefinitely administered to this class of patients, carrying a plethora of side effects.

Nonetheless, this systematic review comes with a number of limitations that could be addressed in further research—most importantly, the lack of standardized protocols concerning treatment frequency and duration. Most authors followed their patients for up to three months, whereas some, such as Yan et al. [32] or Shawky et al. [33], provided a double progress assessment at one and three months. Likewise, Lechien et al. [37] included a follow-up of 10 weeks. Conversely, other authors did not report the exact duration of their follow-up. Generally speaking, patients were not followed up for less than one month, with the shortest follow-up duration being carried out by Steffens et al. [34] and Evman et al. [39].

Secondly, the heterogeneity of the analyzed outcomes made it impossible to conduct a meta-analysis. The TDI score was the most used objective outcome. Yet, other authors, such as Shawky et al. [33], Evman et al. [39], and Tabrizi et al. [19], employed different validated tools, like the Q-Sticks test, CCRC, or the I-SIT, respectively.

Differently, the ODQ score was used by Lechien et al. [37] as a qualitative measure of olfaction. All other reports of subjective olfaction progression were inherently narrative, preventing us from synthesizing and comparing these efficacy measures. Furthermore, outcomes were inconsistently reported across the available studies, where some included solely qualitative outcomes, while others focused strictly on the quantitative side. Finally, the limited number of available studies restricts the generalizability of our findings, as they may not accurately mirror the phenomenon in its entirety. To this day, no guidelines provide a precise timeline of when to intervene in the natural history of anosmia or hyposmia. Of note, there was a tendency to exclude patients with a history of OD lasting less than 6 months since spontaneous recovery still represents a possibility, but also patients with a history longer than 12 months, as peripheral nerve regeneration is unlikely. On top of that, the duration of improvement in olfactory function is also an unknown factor. Further studies involving larger sample sizes and a longer follow-up are needed to corroborate the evidence of PRP being a safe and effective treatment option for OD patients.

## 5. Conclusions

The findings presented in this review have demonstrated the potential of PRP as a secure and reliable therapeutic option for improving OD in a wide variety of patients, including those who have recovered from COVID-19. However, there are only very few short-term recent studies on the effectiveness of PRP treatment in patients with OD. In order to confirm and improve these encouraging results, there is an urgent need for dedicated controlled trials to test the clinical benefits of the PRP in different categories of OD patients and compare the effectiveness of various therapeutic protocols in terms of treatment schedules. This will open up possibilities for PRP to be incorporated into routine clinical practice for OD.

## Figures and Tables

**Table 1:** General characteristics of the included studies.

Author (Year)	Country	Journal	Study Design	N. of Patients	Mean Age (Years)	Sex (M/F)	Type of Olfactory Dysfunction	Duration of Olfactory Dysfunction (Months)	Etiology of Olfactory Dysfunction
**PRP and Olfactory Dysfunction**									
Mavrogeni et al., 2016 [31]	Hungary	International Tinnitus Journal	Prospective	5	49	2/3	Anosmia: 5 patients	>3.0 months	Post-traumatic: 1 patient Post-viral: 4 patients
Yan et al., 2020 [32]	United States	Laryngoscope Investigative Otolaryngology	Pilot study	7	51.1	0/7	Functional anosmia: 2 patients Hyposmia: 5 patients	6.0–11.0 months (8.42 months)	Post-traumatic: 1 patient Post-viral: 5 patients Post-anesthetic exposure: 1 patient
Shawky et al., 2023 [33]	Egypt	Indian Journal of Otolaryngology and Head & Neck Surgery	Prospective	PRP single injection group: 27 PRP double injection group: 27	45.9 ± 10.3	30/24	Anosmia: 54 patients	6.0–11.0 months	
**PRP and COVID-19**									
Steffens et al., 2022 [34]	Belgium	European Archives of Oto-Rhino-Laryngology	Prospective	PRP group: 30 No PRP group: 26	PRP group: 39 ± 12 No PRP group: 44 ± 11	PRP group: 14/16 No PRP group: 6/20	Post-COVID19 chronic olfactory dysfunction: 56 patients	PRP: 10.8 months; control: 9.7 months	COVID-19
El Naga et al., 2022 [35]	Egypt	The Egyptian Journal of Otolaryngology	Pilot study	PRP group: 30 No PRP group: 30	PRP group: 28.9 No PRP group: 30.07	PRP group: 11/19 No PRP group: 9/21	Post-COVID-19 parosmia: 60 patients	>3.0 months	COVID-19
Yan et al., 2022 [36]	United States	International Forum of Allergy Rhinology	RCT	PRP group: 18 No PRP group: 12	PRP group: 44.6 No PRP group: 43.4	PRP group: 9/9 No PRP group: 6/6	Post-COVID-19 olfactory dysfunction: 30 patients	6.0–11.0 months (PRP: 8.6 months; control: 8.9 months)	COVID-19
Lechien et al., 2022 [37]	Belgium	European Archives of Oto-Rhino-Laryngology	Prospective	87	41.6 ± 14.6	25/62	Post-COVID-19 anosmia: 30 patients Post-COVID-19 hyposmia: 40 patients; Post-COVID-19 parosmia:17 patients	15.7 months	COVID-19
Lechien et al., 2023 [38]	Belgium; Italy; France	Otolaryngology-Head and Neck Surgery	Multicenter controlled study	PRP group: 81 No PRP group: 78	PRP group: 43.5 ± 13.4 No PRP group: 47.0 ± 11.1	PRP group: 20/61 No PRP group: 26/52	Post-COVID-19 anosmia: 55 patients Post-COVID-19 hyposmia: 79 patients; Post-COVID-19 parosmia: 25 patients	PRP: 15.7 months; control: 11.0 months	COVID-19
Evman et al., 2023 [39]	Turkey	Revista da Associação Médica Brasileira	RCT	PRP group: 12 No PRP group: 13	PRP group: 31.8 ± 6.9 No PRP group: 33.5 ± 11.1	PRP group: 6/6 No PRP group: 6/7	Post-COVID-19 olfactory dysfunction: 25 patients	>12.0 months	COVID-19
**PRP and Functional Endoscopic Sinus Surgery**									
Tabrizi et al., 2021 [19]	Iran	Medical Journal of the Islamic Republic of Iran	RCT	PRP group: 27 No PRP group: 21	PRP group: 37.2 ± 7.6 No PRP group: 34.4 ± 7.0	PRP group: 19/8 No PRP group: 15/6	Anosmia: 48 patients	6.6 months (PRP: 6.9 months; control: 6.1 months)	Chronic rhinosinusitis

PRP: Platelet-rich plasma.

**Table 2:** Summary of the results obtained in the included studies.

Author (Year)	TDI		Other Objective Outcomes		ODQ		Other Subjective Outcomes
	Baseline	Follow-Up	Baseline	Baseline	Follow-Up	Baseline	
**PRP and Olfactory Dysfunction**							
Mavrogeni et al., 2016 [31]	-	-	-	-	-	-	After the third therapeutic procedure, they could differentiate the smells of daily life.
Yan et al., 2020 [32]	19.5	1 month: 21.2 3 months: 23.1	-	-	-	-	All patients reported a subjective improvement of their smell shortly after injection.
Shawky et al., 2023 [33]	-	-	Q-Sticks test: 100% anosmia	1 month: Q-Sticks test: 100% mild improvement; 3 months: Q-Sticks test and single injection: 40.7% insignificant improvement, 59.3% significant improvement; Q-Sticks test and double injection: 29.6% insignificant improvement, 70.4% significant improvement	-	-	All patients reported subjective improvement of their smell shortly after injection.
**PRP and COVID-19**							
Steffens et al., 2022 [34]	PRP: 21.3 ± 7.4 control: 24.5 ± 7.4	1 month: PRP: 28.0 ± 5.0 control: 25.0 ± 7.7	-	-	-	-	Self-assessment of improvement in smell function: PRP: 1.8 control: 0.3
El Naga et al., 2022 [35]	-	-	-	-	-	-	VAS for parosmia PRP: *p* < 0.00001 Control: *p*: 0.00148
Yan et al., 2022 [36]	PRP: 24.3 control: 26.0	1 month: PRP: 28.6 control: 27.2 3 months: PRP: 30.6 control: 28.6	-	-	-	-	Significant improvement in VAS at 1 and 3 months compared to baseline in both groups. No significant difference in the change of subjective olfaction scores (VAS) between placebo and intervention.
Lechien et al., 2022 [37]	20.3 ± 10.5	2 months: 26.0 ± 11.2	-	-	Parosmia: 7.8 ± 3.8 Life quality statement: 34.1 ± 13.8 Sincerity statement: 9.1 ± 4.4 ODQ total: 51.0 ± 18.0	2 months: Parosmia: 7.5 ± 3.1 Life quality statement: 24.4.1 ± 8.0 Sincerity statement: 8.9 ± 3.3 ODQ total: 40.7 ± 10.9	No subjective improvement of olfactory dysfunction: *n* = 8 substantial improvement of anosmia: *n* = 20 substantial improvement of hyposmia: *n* = 9
Lechien et al., 2023 [38]	PRP: 19.8 ± 9.5 control: 21.5 ± 8.4	10 weeks: PRP: 28.5 ± 9.1 control: 25.4 ± 7.7	-	-	Parosmia PRP: 7.6 ± 3.8 Parosmia control: 7.9 ± 3.7 Life quality statement PRP: 34.1 ± 14.2 Life quality statement control: 30.8 ± 10.7 Sincerity statement PRP: 9.7 ± 4.5 Sincerity statement control: 6.0 ± 2.7 ODQ total PRP: 51.3 ± 18.6 ODQ total control: 44.6 ± 12.5	10 weeks: Parosmia PRP: 6.9 ± 3.3 Parosmia control: 7.4 ± 3.4 Life quality statement PRP: 24.2 ± 9.2 Life quality statement control: 27.1 ± 10.6 Sincerity statement PRP: 7.5 ± 3.4 Sincerity statement control: 11.1 ± 3.9 ODQ total PRP: 38.6 ± 11.7 ODQ total control: 45.5 ± 13.3	65 patients experienced subjective smell improvement after a mean duration of 3.4 ± 1.9 weeks.
Evman et al., 2023 [39]	-	-	STC PRP: 5.6 STC control: 5.7 SIC PRP: 11.4 SIC control: 11.2	1 month: STC PRP: 6.5 STC control: 5.8 SIC PRP: 15.2 SIC control: 11.9	-	-	-
**PRP and Functional Endoscopic Sinus Surgery**							
Tabrizi et al., 2021 [19]	-	-	I-SIT PRP: 5.85 I-SIT control: 5.62	3 months: I-SIT PRP: 18.9 I-SIT control: 18.4	-	-	-

PRP: Platelet-rich plasma. TDI: threshold, discrimination, and identification test. ODQ: VAS for parosmia, olfactory dysfunction questionnaire. -: not available. STC: smell detection threshold. SIC: smell identification test. I-SIT: Iran Smell Identification Test.

## Data Availability

The original contributions presented in the study are included in the article, further inquiries can be directed to the corresponding author.
